# Cigarette Smoke Exposure, Pediatric Lung Disease, and COVID-19

**DOI:** 10.3389/fphys.2021.652198

**Published:** 2021-04-27

**Authors:** Marta Schiliro, Elizabeth R. Vogel, Lucia Paolini, Christina M. Pabelick

**Affiliations:** ^1^Department of Anesthesiology and Perioperative Medicine, Mayo Clinic, Rochester, MN, United States; ^2^Department of Pediatric, San Gerardo Hospital, University of Milano-Bicocca, Fondazione MBBM, Monza, Italy; ^3^Department of Physiology and Biomedical Engineering, Mayo Clinic, Rochester, MN, United States

**Keywords:** cigarette smoke exposure, E-cigarette, vaping, lung, coronavirus disease 2019, pediatric, infection, inflammation

## Abstract

The detrimental effects of tobacco exposure on children’s health are well known. Nonetheless, the prevalence of secondhand or direct cigarette smoke exposure (CSE) in the pediatric population has not significantly decreased over time. On the contrary, the rapid incline in use of e-cigarettes among adolescents has evoked public health concerns since increasing cases of vaping-induced acute lung injury have highlighted the potential harm of these new “smoking” devices. Two pediatric populations are especially vulnerable to the detrimental effects of cigarette smoke. The first group is former premature infants whose risk is elevated both due to their prematurity as well as other risk factors such as oxygen and mechanical ventilation to which they are disproportionately exposed. The second group is children and adolescents with chronic respiratory diseases, in particular asthma and other wheezing disorders. Coronavirus disease 2019 (COVID-19) is a spectrum of diseases caused by infection with the severe acute respiratory syndrome coronavirus 2 (SARS-CoV-2) that has spread worldwide over the last year. Here, respiratory symptoms ranging from mild to acute respiratory distress syndrome (ARDS) are at the forefront of COVID-19 cases among adults, and cigarette smoking is associated with worse outcomes in this population, and cigarette smoking is associated with worse outcomes in this population. Interestingly, SARS-CoV-2 infection affects children differently in regard to infection susceptibility, disease manifestations, and complications. Although children carry and transmit the virus, the likelihood of symptomatic infection is low, and the rates of hospitalization and death are even lower when compared to the adult population. However, multisystem inflammatory syndrome is recognized as a serious consequence of SARS-CoV-2 infection in the pediatric population. In addition, recent data demonstrate specific clinical patterns in children infected with SARS-CoV-2 who develop multisystem inflammatory syndrome vs. severe COVID-19. In this review, we highlight the pulmonary effects of CSE in vulnerable pediatric populations in the context of the ongoing SARS-CoV-2 pandemic.

## Introduction

The detrimental effects of cigarette smoke exposure (CSE) on pediatric respiratory function have been well demonstrated (Jaakkola and Jaakkola, 1997; Weiss et al., 1999; Walker et al., 2003; Jackson et al., 2014; Liptzin et al., 2015). Although the number of cigarette smokers continues to decrease, new smoking devices (e-cigarettes) have spread among adolescents^1^ carrying with them the perception that they are “safer” than traditional tobacco products. This is especially concerning, since it is now well demonstrated that use of e-cigarettes is associated with subsequent use of traditional cigarettes, and vaping itself can cause significant lung injury, particularly among teenagers.^2^

Cigarette smoke exposure in children not only leads to wheezing, recurrent pulmonary and ear infections, and asthma, but it may also worsen pre-existing lung conditions (Weiss et al., 1999; Walker et al., 2003; Jackson et al., 2014). Here, former premature babies who suffer from chronic lung diseases following oxygen therapy and/or respiratory support are at increased risk. Similarly, children with asthma or chronic wheezing disorders often experience recurrent exacerbations and poor disease control when exposed to cigarette smoke.

The ongoing severe acute respiratory syndrome coronavirus 2 (SARS-CoV-2) pandemic has dramatically challenged healthcare systems and daily life worldwide and its ultimate impact on children affected by chronic lung conditions remains to be seen. At the beginning of the outbreak, few cases and less severe manifestations with low mortality rates were described in children (Chang et al., 2020), suggesting that adults, especially the elderly, were predominantly affected. Initially, children were largely considered a significant source of spread but not a population that would be critically impacted by infection. However, as the pandemic has unfolded, a shift toward the younger population has been reported in the United States (Boehmer et al., 2020) and Europe^3^, and children’s unique infection susceptibility, disease manifestations, and health consequences are increasingly appreciated.^4^ In addition, societal restrictions imposed by the health crisis are challenging children’s daily life, education, and socialization opportunities, thereby impacting the management of their chronic medical conditions. This is particularly true in lower-income settings. Currently, the long-term consequences of coronavirus disease 2019 (COVID-19) for children’s health remain difficult to foresee.

With this review, we attempt to highlight the detrimental effects of CSE in the pediatric population with particular focus on vulnerable children, including premature babies and children with pre-existing lung conditions, and to discuss the potential interplay of SARS-CoV-2 infection in these settings.

## How Big is the Problem?

### Cigarette Smoke Exposure in Children and Adolescents

Cigarette smoke derives from burning tobacco products (e.g., cigarettes, cigars, and pipes) or smoke that has been exhaled by a person smoking. Secondhand smoke (SHS) exposure represents the typical source of CSE in children. In the US, more than 128 million nonsmokers are exposed to SHS at any age (Best et al., 2009). Children, however, have a higher risk of SHS exposure compared to the nonsmoking adult population because of their frequent exposure from household members (Gergen et al., 1998; Pirkle et al., 2006). The World Health Organization estimates that around 700 million children – almost half of the world’s pediatric population – have significant exposure to SHS.

SHS exposure in children has a strong impact on their lifelong health and places a significant burden on the health care system. In the United States in 2010, exposure to SHS in children caused 101,570 visits to the emergency department with a total of 62.9 million dollars spent (Yao et al., 2019). Analysis from the Global Burden of Disease Study 2017 estimated that 63,822 pediatric (<10 years of age) deaths worldwide were ascribable to SHS exposure that year (GBD 2017 Risk Factor Collaborators, 2018).

Detrimental effects on respiratory function are common in children exposed to SHS. A systematic review and meta-analysis of 60 studies concluded that passive smoke exposure increases the risk of pediatric lower respiratory tract infections, with the risk becoming even greater in cases where both parents smoke (OR single smoking parent 1.22–95% Cl 1.10–1.35 – vs. OR both smoking parents 1.54–95% Cl 1.40–1.69; Jones et al., 2011). Not only does SHS exposure facilitate the onset of respiratory diseases and infections, but it may also exacerbate symptoms of pre-existing diseases such as asthma.

Airway diseases such as wheezing and asthma are the most common chronic respiratory disorders in the pediatric population and may have significant lifelong impact on children’s health, even spanning into adulthood (von Mutius, 2001; Tai et al., 2014; Um-Bergstrom et al., 2019). According to the Center for Disease Control and Prevention (CDC)^5^, 5.5 million children under age 18 in the United States are currently affected by asthma, and 7.1 million suffer from respiratory allergies. CSE – both direct and secondhand – enhances the risk of developing asthma in infancy, childhood, and adolescence (Goksor et al., 2007; Burke et al., 2012; Thacher et al., 2014, 2018). A systematic review by Burke et al. (2012) showed that SHS exposure in children and young adults increases the incidence of asthma by 20%. Moreover, SHS exposure is associated with more severe asthma symptoms (Akinbami et al., 2013; Hollenbach et al., 2017), increased risk of asthma exacerbations (Wang et al., 2015; Neophytou et al., 2018), and hospitalizations (Das, 2003; Wang et al., 2015).

Within the last decade, the introduction of e-cigarettes and vaping has evoked significant public health concerns, specifically in regard to youth. In fact, the number of adolescents who vape has constantly and significantly increased during the past few years (Centers for Disease Control and Prevention, 2013; Carroll Chapman and Wu, 2014; Miech et al., 2019). In 2019, over 5 million high schoolers (27.5%) in the United States reported using e-cigs in the past 30 days, and, alarmingly, teenagers with asthma are even more likely than their non-asthmatic peers to try e-cigarettes (Choi and Bernat, 2016; Fedele et al., 2016; Larsen et al., 2016; Kim et al., 2017; Reid et al., 2018; Turner et al., 2018; Martinasek et al., 2019). Moreover, initial use of e-cigarettes during adolescence is strongly associated with subsequent traditional cigarette smoking, contributing to the increase of smoking in the adult population. Since March of 2019, outbreaks of e-cigarette and vaping product use-associated lung injury (EVALI) have been reported throughout the United States, in particular among adolescents^2^ (Layden et al., 2020). EVALI is a clinical syndrome that comprises constitutional, respiratory, and gastrointestinal symptoms associated with inflammatory response and pulmonary infiltrates in patients who have recently used (within 90 days) e-cigarette products, and that is not ascribable to any other cause. EVALI is most associated with cannabinoid vaping and vitamin E acetate containing products^2^; however, the mechanisms responsible for vaping-induced lung injury are still under investigation. COVID-19 and EVALI have similar presenting symptoms. Therefore, a high level of suspicion for EVALI is still recommended during the COVID-19 pandemic since the possible overlap of clinical manifestations between COVID-19 and EVALI may lead to late EVALI diagnosis and treatment. This is highlighted by some recently reported clinical case series (Darmawan et al., 2020; Hassoun et al., 2020; Anderson et al., 2021).

### COVID-19 in Children

The American Academy of Pediatrics together with The Children’s Hospitals Association provides a “Weekly State Data Report” to trace COVID-19 cases in children in the United States.^6^ As of December 31, 2020, children represent 12.4% of all the United States COVID-19 cases (2,128,587/17,137,295). Of significance, pediatric COVID-19 cases have constantly increased over the last few months (17% increases in child cases over the last 2 weeks 12/17-12/31/2020). Hospitalizations and deaths are fortunately uncommon in United States children: between 0.2 and 3.4% of all pediatric COVID-19 cases resulted in hospitalization and 0.00 and 0.08% of all pediatric COVID-19 cases resulted in death. Similar trends are reported in Europe.^7^

Children of all ages are susceptible to SARS-CoV-2 infection, although most infected children are asymptomatic (Bellino et al., 2020; Davies et al., 2020; Dong et al., 2020; Parri et al., 2020). When symptomatic, children present with different clinical manifestations, laboratory, and radiological findings compared to adult patients (Verma and Amin, 2020; Xia et al., 2020). The incubation period is like adults, 2–14 days with an average of 6 days.^8^ Pediatric patients typically have a milder disease course overall. Significantly, children from low-income settings represent a high proportion of pediatric hospitalized patients; however, this seems not to be consistently related to worse outcome (Fernandes et al., 2021).

Presenting symptoms are mostly non-specific and self-limiting, which makes it difficult to distinguish COVID-19 from other common viral illnesses, possibly driving a lower testing rate in this population. Children may or may not have fever, and usually present either with cough or gastrointestinal symptoms in addition to more general symptoms such as rhinorrhea, sore throat, headache, and myalgia (Verma and Amin, 2020; Yoon et al., 2020; Fernandes et al., 2021). Of note, 40–50% of COVID-19 pediatric cases have a documented coinfection with other respiratory pathogens (Wu et al., 2020). Inflammatory markers are commonly not elevated in mild cases (Verma and Amin, 2020). Chest CT findings in pediatric COVID-19 cases with respiratory involvement showed unilateral or bilateral subpleural ground-glass opacities and consolidations with surrounding halo sign (Xia et al., 2020).

Since April of 2020, cases of children becoming severely ill after SARS-CoV-2 infection with features resembling Kawasaki disease have been increasingly reported^9^ (Riphagen et al., 2020; Verdoni et al., 2020). This severe clinical manifestation has been named multisystem inflammatory syndrome in children (MIS-C) associated with COVID-19. Initial CDC criteria^10^ for MIS-C included “(1) persistent fever ≥38.0°C; (2) laboratory evidence of inflammation; (3) evidence of clinically severe disease requiring hospitalization; (4) multisystem (≥2) organ involvement; (5) positive for current or recent SARS-CoV-2 infection; and (6) no alternative plausible diagnoses.” It has become clear that MIS-C is a separate clinical and possibly pathological, entity from severe pediatric SARS-CoV-2 (Jiang et al., 2020). Interestingly, MIS-C cases lagged the COVID-19 case curve by 1 month and only one third of children diagnosed with MIS-C had RT-PCR positivity for SARS-CoV-2 depicting an active infection, while most of them were antibody positive (Jiang et al., 2020). A recent case series of 1,116 hospitalized pediatric patients from United States surveillance data has provided insights into the clinical characteristics and outcomes of children and adolescents with MIS-C compared with severe COVID-19 (Feldstein et al., 2021). Patients with MIS-C were more likely to be age 6–12 years, non-Hispanic black race, presenting severe cardiovascular or mucocutaneous involvement, and more severe inflammation. On the other hand, preliminary reports suggested that severe pediatric COVID-19 cases were more frequent in younger infants (<1 year of age; Gotzinger et al., 2020; Liu et al., 2020; Yang et al., 2020a), however, recent reports did not confirm this trend. COVID-19 severity in children seems to be associated with age ≥ 10 years, hypoxemia, obesity, and one or more underlying medical conditions (Bellino et al., 2020; Dong et al., 2020; Shekerdemian et al., 2020; Feldstein et al., 2021; Fernandes et al., 2021; Ouldali et al., 2021).

## Pathophysiologic Insights of Cigarette Smoke‐ and SARS-CoV-2-Induced Lung Inflammation

**Figure 1:**
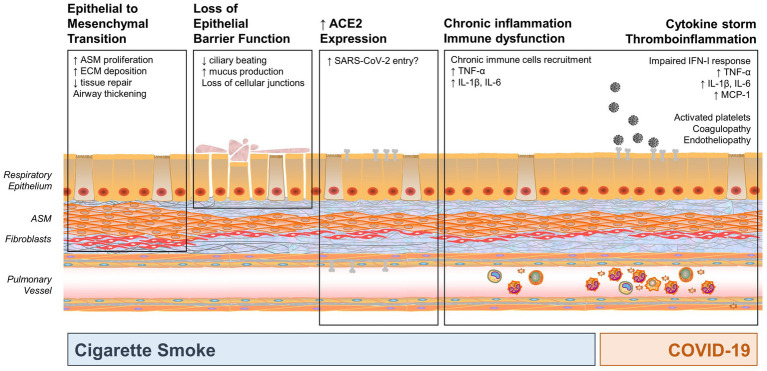
Mechanisms by which cigarette smoke exposure (CSE) and coronavirus disease 2019 (COVID-19) may impact the developing and pediatric airway, highlighting potential areas of synergy. CSE results in three main impacts: epithelial to mesenchymal transformation, loss of epithelial barrier function (which may increase susceptibility to infection), and chronic inflammation and immune dysfunction. In addition, smoking increases angiotensin-converting enzyme 2 (ACE2) expression, which is a key entry point for severe acute respiratory syndrome coronavirus 2 (SARS-CoV-2), thus potentially increasing susceptibility to infection. COVID-19 also results in significant inflammation and cytokine response. Synergy between CSE and SARS-CoV-2 may occur in activation of pro-inflammatory mediators, such as TNF-α, IL-1β, IL-6, and other mediators that may result in cytokine storm, pronounced inflammatory state, and immune dysfunction. (ASM, airway smooth muscle; ECM, extracellular matrix).

As we learn more about the effects of SARS-CoV-2 in children, the question arises whether and how cigarette smoke and SARS-CoV-2 represent a new intertwined threat for children with underlying lung disease. There is concern about the possible “second hit” effect of COVID-19 on developing lungs that are already chronically impacted by environmental factors and/or prematurity and its consequences. Understanding the pathophysiologic mechanisms of CSE and SARS-CoV-2 induced inflammation and lung injury demonstrates potential areas of overlap or synergism that may lead to long-term pulmonary consequences in vulnerable pediatric populations. In this section, we review some of those mechanisms and their impact on pulmonary inflammation and injury, as summarized in Figure 1.

### Cigarette Smoke and Vaping

Tobacco smoke contains more than 7,000 chemicals, including hundreds that are toxic and about 70 that can cause cancer. Tobacco burning releases a complex mixture of chemicals, both in gaseous and particulate form, that have cytotoxic and mutagenic properties, along with antigenic capacities that contribute to chronic inflammation (Lee et al., 2012). Oxidative imbalance and stress caused by CSE are responsible for direct genetic or epigenetic effects that lead to altered barrier function of the epithelial layer, epithelial to mesenchymal transition (EMT), and immune dysfunction.

Due to their anatomic location, the epithelial layers of the oral cavity and airways act as the first barrier to cigarette smoke’s effects and are the cornerstone of cigarette smoke-induced inflammatory response. In regard to epithelial barrier function, cigarette smoke impairs mucociliary clearance by reducing ciliary beating and enhancing mucus production. Moreover, cigarette smoke derivatives disrupt epithelial cellular junctions, allowing for a deeper penetration of toxins and infectious agents (Aghapour et al., 2018).

Epithelial to mesenchymal transition is associated with redox imbalance and oxidative stress (Milara et al., 2013; Gohy et al., 2015) and represents an important consequence of cigarette smoke effects. EMT of airway epithelium promotes airway wall thickening and remodeling, thereby leading to airway obstruction (Eurlings et al., 2014). Alveolar EMT, rather, impacts re-epithelization (Eurlings et al., 2014; Shen et al., 2014) and contributes to the development of emphysema (Aghapour et al., 2018). EMT leads to increased fibroblast migratory capacity, invasiveness, resistance to apoptosis, and greatly increased production of extracellular matrix (ECM) components (Kalluri and Neilson, 2003).

Another important consequence of cigarette smoking is immune dysfunction. Epithelial and innate immune cells are highly responsive to cigarette derivates. Oxidative stress triggers the activation of transcription factors involved in inflammatory responses, such as NF-kB and AP-1. Under the activation of these transcription factors, CSE promotes a huge production of proinflammatory mediators (e.g., IL-1β, IL-6, TNF-α, and granulocyte/monocyte CSF) and chemokines that are responsible for sustained immune cell recruitment and activation (Lee et al., 2012). This hyperinflammatory environment contributes to enhanced reactivity to inhaled antigens, tissue damage, and remodeling. Of note, despite being hyperactivated, innate and adaptive immune responses are highly dysfunctional in this setting (Lee et al., 2012). AP-1 activation has been related to corticosteroid resistant inflammation (Walters et al., 2005). Constitutively activated inflammation pathways are associated with reduced response to acute infectious challenges. Similarly, immune responses to viral antigens are significantly reduced, as cigarette smoke downregulates Toll-like receptor (TLR) 3-mediated responses to double-stranded RNA (Todt et al., 2013). Cigarette smoke-induced oxidative stress impairs the phagocytic activity of alveolar macrophages leading to accumulation of cellular debris and the initiation of necrotic processes. In regard to T-cells adaptive immune responses, cigarette smoke suppresses T helper (Th) 1 activation and enhances Th2 and Th17 inflammation that have been associated with eosinophilic inflammation, epithelial dysfunction, and virus-induced exacerbations in COPD patients (Papi et al., 2006).

Furthermore, CSE directly influences airway smooth muscle (ASM) function, enhancing proliferation, ECM deposition, and mitochondrial dysfunction (Aravamudan et al., 2014, 2017; Vogel et al., 2014).

The pathophysiology associated with EVALI is only beginning to emerge, but among the clinical manifestations are inflammation, wet cough, phlegm, and mucociliary dysfunction (Hedman et al., 2018; Chung et al., 2019). Data suggest that e-cigarette users produce significantly more sputum than smokers and show increased markers of inflammation in their airways. As airway inflammation and mucus hypersecretion are central features underlying asthma pathology, asthmatics might be at increased risk for e-cigarette-induced health effects (Kim et al., 2017).

### SARS-CoV-2 Infection

Severe acute respiratory syndrome coronavirus 2 infection starts with the viral spike protein (S-protein) binding to angiotensin-converting enzyme 2 (ACE2) receptors on the cell surface (Walls et al., 2020). ACE2 is largely expressed in epithelial cells of the respiratory tract, vascular endothelial cells, and alveolar monocytes and macrophages (Lu et al., 2020). For SARS-CoV-2 to enter cells, the S-protein must be cleaved at two different sites by host cell proteases Furin and transmembrane serine protease 2 (TMPRSS2). This process promotes cell membrane fusion and internalization of the virus. Throughout the whole process of virus contact, internalization, and replication, infected host cells are provided with a series of pattern recognition receptors (PRRs) that recognize pathogen-associated molecular patterns (PAMPs) and allow the antiviral response to start. SARS-CoV-2’s lipids, proteins, genetic materials, together with intracellular calcium homeostasis alterations, are recognized by PRR as TLRs 3, 4, 7, 8, and 9, RIG-I, and MDA-5. Viral recognition leads to activation of various inflammatory pathways as NLRP3 inflammasome that induces proinflammatory cytokine release, caspase activation, and cell death; MAPK pathway leads to IL-1β/2/6/10/18, and TNF*α*; secretion of interferon (IFN)-α/β (Kumar et al., 2021). INFs are key players in anti-viral host responses through inhibition of virus replication and immune activation. Interestingly, SARS-CoV-2 can inhibit an early type I IFN response in infected cells (Arunachalam et al., 2020; Miorin et al., 2020). This suppressed IFN-I production allows higher viral replication and tissue damage, further enhancing lung inflammation (Brodin, 2021). Bronchial epithelial cells, type I and II alveolar epithelial cells, and capillary endothelial cells are the site of primary infection leading to immune cell recruitment into the alveolar space and cytokine release. Many cases of severe COVID-19 are characterized by fever, elevated acute phase response markers, coagulopathy, and hemophagocytosis, suggesting a role for lung-derived cytokine storm in the pathogenesis of acute respiratory distress syndrome (ARDS), immunothrombosis, and multi-organ dysfunction (Fajgenbaum and June, 2020; Mcgonagle et al., 2021). Recent data demonstrate the persistence of anti-spike IgG neutralizing antibodies up to several months after the initial infection in >90% of infected individuals (Gudbjartsson et al., 2020; Wajnberg et al., 2020).

The significant variability in the clinical course of SARS-CoV-2 infections – ranging from asymptomatic infection, cold-like symptoms, to severe ARDS and multi-organ failure – has prompted the scientific community to identify determinants of disease severity. Male sex, age, and pre-existing conditions, such as hypertension, diabetes, and obesity are poor prognostic factors in COVID-19 (Sanyaolu et al., 2020). Impaired type I IFN responses, T_H_1 > T_H_2 cell response, high neutrophil-to-lymphocyte ratio, pre-existing inflammatory states associated with aging, and chronic diseases have been described as associated with worse outcomes (Del Valle et al., 2020; Brodin, 2021).

Focusing on children, lower ACE2 gene expression has been reported in the nasal epithelium of younger children (4–9 years old) which may in part explain the lower susceptibility to SARS-CoV-2 infection in children (Molloy and Bearer, 2020). On the other hand, children may rely on a more robust innate immune response (Consiglio et al., 2020; Gruber et al., 2020; Pierce et al., 2020) that allow them to limit viral replication and experience milder infection.^11^ The higher number of naïve T-cells in children (Kumar et al., 2018), as well as a more recent memory for other coronaviruses infections (Mateus et al., 2020) have also been implicated in the difference in infections. There are also reports describing a different antibody response in the pediatric population regardless of disease severity (Weisberg et al., 2021). Independently from MIS-C development, SARS-CoV-2-infected children produce predominantly IgG anti-S antibody, with lower titers of anti-S IgM and IgA compared to adults. Moreover, children show significantly lower anti-N IgG that may be consistent with a lower viral load (Weisberg et al., 2021). The other aspect that has puzzled the scientific community is MIS-C pathophysiology. The temporal relationship between COVID-19 and MIS-C cases suggests that it is not directly related to viral infection but rather to the development of adaptive immunity. The massive cardiovascular involvement suggests an underlying immune-mediated disease, and proposed mechanisms involve the production of autoantibodies as a result of viral mimicry of the host, T-cells recognition of viral antigens on infected cells, and immune complexes (Jiang et al., 2020).

### COVID-19 and Smoking

Smoking has definitely been associated with COVID-19 progression and worse outcomes in adults (Gulsen et al., 2020; Patanavanich and Glantz, 2020; Vardavas and Nikitara, 2020; Zhao et al., 2020). COPD patients, who typically have a significant history of smoking, are at increased risk for severe COVID-19 (Zhao et al., 2020). ACE2 gene expression in airway epithelium is upregulated by tobacco smoking (Cai et al., 2020; Leung et al., 2020), possibly through the α7 subtype of the nicotine acetylcholine receptor (Russo et al., 2020). Because ACE2 receptors are an important entry point for COVID-19, this may represent an increased risk for viral entry and infection in smokers. Significantly, a recent *in vitro* study demonstrated that acute CSE induced increased infection severity in air-liquid interface cultures derived from human airway basal stem cells by impairing IFN responses and altering tissue repair (Purkayastha et al., 2020).

Cigarette smoking is associated with underlying chronic inflammation, oxidative stress, and epithelial and immune dysfunction that may also contribute to COVID-19 progression (Gulsen et al., 2020; Shastri et al., 2021). Even if not extensively tested, the same increased risk may apply to SHS and nicotine vapers.

## Pediatric Lung Diseases, Cigarette Smoke Exposure, and COVID-19: a Potential Interplay?

As described above, the overarching impact of cigarette smoke, e-cigarettes, and COVID-19 is a robust inflammatory pulmonary response. While cigarette smoke-induced inflammation is meant to serve as a natural/protective response, it may make children more vulnerable to illness, since the inflammatory mediators associated with CSE may result in exacerbation of inflammatory and remodeling pathways typical of chronic pediatric lung disease. This vulnerability in combination with an immature immune system – depending on the age of the child – serves as an additional insult to the patients’ respiratory and general health. While the specific contribution of COVID-19 to pediatric lung disease remains to be fully elucidated, other respiratory infections have been shown to exacerbate pediatric lung disease by increasing activation of inflammatory pathways. These multiple “hits” of CSE and pulmonary infections may certainly exacerbate preexisting chronic pediatric pulmonary disease or lead to the development of pulmonary disease in at-risk children.

### Wheezing Disorders, Cigarette Smoke Exposure, and COVID-19

Children with wheezing disorders represent a pediatric population at particular risk from additional “hits” such as CSE and respiratory infections. Asthma and other wheezing disorders present a heterogeneous spectrum characterized by chronic airway inflammation, airway hyperresponsiveness, and airway remodeling (Homer and Elias, 2005; Siddiqui and Martin, 2008; Bush and Menzies-Gow, 2009; Britt et al., 2013; Bonato et al., 2019). Clinically, asthma presents with respiratory symptoms, such as wheezing, shortness of breath, chest tightness, and cough, which cause variable expiratory flow limitations. These symptoms and their intensity can vary over time and may be induced by superimposed respiratory infections or environmental exposures (Deshpande and Morgan, 2016; Testa et al., 2020). Atopy, allergic diseases such as allergic rhinitis, and maternal asthma are all risk factors increasing the likelihood of airway hyperreactivity and asthma (Martinez et al., 1995; Savenije et al., 2011). In fact, even in utero smoke exposure (maternal smoking) has been shown to increase the likelihood of developing asthma in vulnerable children (Liptzin et al., 2015). SHS exposure increases the risk of developing asthma and exacerbation of asthma symptoms (Jaakkola and Jaakkola, 1997; Weiss et al., 1999; Walker et al., 2003; Jackson et al., 2014). Indeed, chronic SHS exposure is known to exacerbate asthma by contributing and/or further enhancing airway hyperresponsiveness and structural changes (Aravamudan et al., 2017). Here, it seems that structural changes due to increased ASM proliferation and increased airway contractility due to enhanced intracellular calcium response play a major role (Hartman et al., 2012; Vogel et al., 2014).

In regard to COVID-19 and pediatric wheezing disorders, a cross-sectional study (Shekerdemian et al., 2020) conducted in North-America’s pediatric intensive care units (PICU) in May 2020 found that, among children with COVID-19 infections that required PICU admission, 80% had comorbidities such as developmental delay and/or genetic anomalies, but only 4% were reported as suffering from chronic lung disease. Overall PICU mortality was reported as <5%. Interestingly, asthma is rarely reported as comorbidity in pediatric COVID-19 cases although asthma is the most common chronic respiratory disease in children. This lack of overlap between COVID-19 and asthma is particularly interesting, as respiratory infections are typically a significant source of asthma exacerbation and morbidity. A systematic review of the current literature (Castro-Rodriguez and Forno, 2020) revealed no current data on the impact of COVID-19 in children with asthma. However, given the potentially significant implications of COVID-19 in children with asthma, the CDC emphasized the positive impact mask wearing and social distancing can make in this vulnerable patient population. Right now, health communities are encouraged to study affected populations in more detail to answer questions about the impact of COVID-19 on children with asthma, asthma severity, and the potential effects of asthma medications in treatment of COVID-19 infections (Castro-Rodriguez and Forno, 2020).

### Prematurity, Cigarette Smoke Exposure, and COVID-19

Infants born prematurely are a second group at particularly high risk of pulmonary morbidity and mortality due to the consequences of interrupting normal prenatal pulmonary development and maturation (Pramana et al., 2011; Vrijlandt et al., 2013; Been et al., 2014). The extent and form of pulmonary compromise varies greatly depending on the level of prematurity and any additional perinatal risk factors to which the infant is exposed (Britt et al., 2013). Extremely preterm infants [<28 weeks gestational age (GA)] have the highest rate of pulmonary insult because they are born prior to the saccular stage of lung development, before even primitive alveoli have started to form. These infants typically present with bronchopulmonary dysplasia (BPD), characterized by alveolar simplification, dysmorphogenesis of the alveolar capillaries, ASM proliferation, and abnormal ECM deposition (Jobe, 1999). Late preterm infants (33–36 weeks GA) are much less likely to develop BPD but are at increased risk of developing reactive airway diseases such as wheezing and asthma (Martin et al., 2013; Mcevoy et al., 2013).

Due to the pulmonary compromise that attends preterm birth, these infants commonly require respiratory support in the form of supplemental oxygen, mechanical ventilation, or continuous positive airway pressure (CPAP) to prevent hypoxia and maintain adequate alveolar recruitment and ventilation. Unfortunately, these necessary therapies may result in unintentional exacerbation of the pulmonary insults of prematurity.

In light of the numerous pulmonary insults to which preterm infants are commonly exposed in the perinatal period, they are particularly susceptible to the addition of environmental insults, such as cigarette smoke and infection. CSE is a well-documented risk factor for development of respiratory disease in the pediatric population (Carlsen and Carlsen, 2008; Vanker et al., 2017; Grant et al., 2020). From the perspective of prematurity, maternal tobacco use is clearly associated with increased risk for premature birth as well as placenta previa, placental abruption, intrauterine growth restriction, and premature rupture of membranes (Andres and Day, 2000). Preterm infants with BPD who are exposed to second-hand smoke have been found to require supplemental oxygen for longer periods of time and to be more likely to require steroid treatment as neonates (Collaco et al., 2014; Martinez et al., 2015).

The impact of COVID-19 on preterm infants is an emerging area of investigation. SARS-CoV-2 infection appears to increase risk of preterm birth and is associated with increased risk of admission to the neonatal intensive care unit (NICU; Allotey et al., 2020; Golden and Simmons, 2020; Mimouni et al., 2020; Pettirosso et al., 2020; Woodworth et al., 2020; Yang et al., 2020b). Intriguingly, one study found that mothers hospitalized with COVID-19 earlier in pregnancy (23–33 weeks GA) were less likely to delivery early than those who were infected later in pregnancy (34–36 weeks GA; Gulersen et al., 2020). In regard to prenatal vertical transmission, there have been case reports of neonates with respiratory symptoms who tested positive for SARS-CoV-2 within 24 h of birth, raising concern for possible vertical transmission (Rivera-Hernandez et al., 2020; Sisman et al., 2020). However, in the vast majority of cases, infants born to mothers with COVID-19 do not test positive or develop symptoms of infection, indicating that if vertical transmission does occur, it is very rare (Pettirosso et al., 2020; Woodworth et al., 2020). Indeed, one study found that postnatal transmission between SARS-CoV-2 positive mothers and infants was very low, even when breastfeeding and allowing the infants to room with their mother after birth. In a series of 120 cases, 83% of the infants roomed with their mother after birth with the majority breastfeeding. All neonates were negative for SARS-CoV-2 after birth. About 96% of these infants were retested for SARS-CoV-2 at 5–7 days of life and none tested positive (Salvatore et al., 2020). In neonates, COVID-19 most often presents with fever and mild respiratory symptoms, though it may present with gastrointestinal symptoms and abdominal distension in a subset of neonates (Golden and Simmons, 2020; Karabay et al., 2020; Marin Gabriel et al., 2020; Ng et al., 2020; Smith et al., 2020). However, there have been case reports of COVID-19 presenting with severe ARDS in the neonatal population (Frauenfelder et al., 2020; Kalyanaraman et al., 2020; Trieu et al., 2020; Wardell et al., 2020).

There is no data at this time evaluating the overlapping exposures of CSE and SARS-CoV-2 in premature infants. Both secondhand cigarette smoke and respiratory infections have been previously shown to act as additional perinatal “hits” to the vulnerable preterm lung that increase the risk of development of chronic lung disease later in life. It is therefore possible that infants infected with SARS-CoV-2 early in life may sustain lasting consequences from this pulmonary insult.

## Summary and Conclusions

Overall, pediatric lung diseases remain a significant source of morbidity and mortality in children. Although advances have been made in the treatment of wheezing disorders and prematurity-related lung diseases, the health care burden remains high. The pathogenesis of such chronic lung conditions is complex and multifactorial. It is now clear that environmental insults play a role increasing susceptibility to pediatric asthma and wheezing disorders and worsen pre-existing lung diseases.

Among the environmental factors that affect the developing lung, cigarette smoke is certainly one of the most prevalent despite being preventable. While the number of adult smokers and the consequent risk of SHS exposure have decreased over time, the introduction of e-cigarettes and other vaping devices has brought up new health concerns such as EVALI and increased susceptibility to subsequent traditional smoking among adolescents. The long-term effects of CSE are ascribable to chronic inflammation and immune disfunction that lead to tissue remodeling and fibrosis, infectious susceptibility, and eventually lung function decline.

Severe acute respiratory syndrome coronavirus 2 represents a new addition to the vast number of infectious diseases that may affect children, and the current “pandemic dimension” makes COVID-19 a very prevalent threat. The clinical spectrum of COVID-19 in children is wide, ranging from asymptomatic to severe respiratory compromise and multisystem inflammatory syndrome. Determinants of COVID-19 clinical syndromes among the pediatric population are still under investigation and new contributing factors continue to emerge as we move through the pandemic. The varied manifestations suggest a role for genetic or acquired predisposition to more severe inflammatory responses. The long-term impact of SARS-CoV-2 infection on respiratory function of children with or without underlying lung diseases remains to be seen. Epidemiological data are key to identifying possible contributing factors and to guide research to better understand COVID-19 pathogenesis.

**Figure 2:**
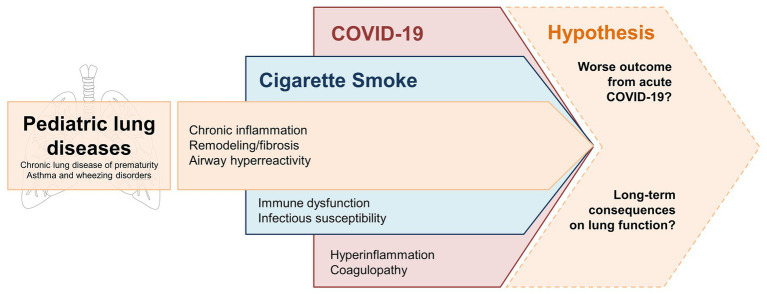
Potential “multiple hits” effect of CSE and COVID-19 on developing lungs affected by common pediatric lung diseases – i.e., chronic lung disease of prematurity, asthma, and wheezing disorders. Underlying processes of inflammation and tissue remodeling are enhanced by chronic CSE. SARS-CoV-2 infection triggers an acute inflammatory response that may compromise the respiratory function of these vulnerable patients. Longitudinal clinical data are needed to confirm whether these factors have an additive effect that leads to more severe COVID-19 manifestations and/or long-term consequences in terms of lung function decline.

In this review, we wanted to highlight the possible detrimental interplay between the chronic inflammatory environment induced by cigarette smoke and the hyperinflammatory stimulus of COVID-19. As outlined in Figure 2, this combination has the potential to exacerbate pulmonary injury in developing lungs already affected by underlying conditions, such as chronic lung disease of prematurity, asthma, and wheezing disorders. While evidence regarding the intersection of cigarette smoke and COVID-19 pulmonary effects remain limited at this time, we agree with recommendations coming from major health associations to strongly encourage cigarette smoking cessation during the ongoing SARS-CoV-2 pandemic^12^ (Ahluwalia et al., 2020). Children and adolescents with pre-existing lung conditions represent an extremely vulnerable population, in particular during this unprecedented time of reduced access to healthcare resources, stay-at-home mandates that may increase the risk of SHS exposure from household members, and social isolation that may encourage addictive behaviors such as vaping in young people.

## Author Contributions

All authors contributed to the manuscript equally and were involved throughout the entire process.

### Conflict of Interest

The authors declare that the research was conducted in the absence of any commercial or financial relationships that could be construed as a potential conflict of interest.

## Footnotes

^1^National Institute of Health (2018)

^2^Center for Disease Prevention and Control (2020)

^3^European Center for Disease Prevention and Control (2020)

^4^AAP (2020a)

^5^CDC (2020a)

^6^AAP (2020b)

^7^European Center for Disease Prevention and Control (2021)

^8^CDC (2020b)

^9^NYC Health (2020)

^10^CDC (2020c)

^11^Nature News (2020)

^12^WHO (2020) and CDC (2020d)
